# Modeling the Greenhouse Gases Data Series in Europe during 1990–2021

**DOI:** 10.3390/toxics11090726

**Published:** 2023-08-24

**Authors:** Alina Bărbulescu

**Affiliations:** Department of Civil Engineering, Transilvania University of Brașov, 5 Turnului Str., 900152 Brasov, Romania; alina.barbulescu@unitbv.ro

**Keywords:** greenhouse gases (GHGs), clustering, regional series, representative temporal series

## Abstract

Nowadays, climate change and atmospheric pollution are two of humanity's most significant challenges. Greenhouse gases (GHGs) are responsible for climate change, and they create effects that are mostly irreversible. Therefore, monitoring and reducing such emissions are compulsory for the preservation of the environment for future generations. The European Union took action in this direction. The article presents the evolution of the total GHGs trend, from 1990 to 2021, in the EU countries and their associates. Trend analysis and grouping of the countries using different clustering techniques are performed. The analysis of the existence of greenhouse gases (GHGs) series’ trend, in 30 countries from Europe, showed that the GHG emissions decreased from 1990 to 2021 in only 17 countries. The annual series, built using the data reported by each country each year, does not present a specific trend. After grouping the countries in clusters by k-means and hierarchical clustering, the representative series for the annual recorded values in the 30 studied countries, called Regional series (RegS), is built using series selected from the cluster with the highest number of elements. The same algorithm provides the Representative Temporal series (TempS), which selects specific years after clustering the annual GHG series.

## 1. Introduction

The climate change impact on the environment, due to the continuous growth of greenhouse gas (GHG) emissions that produce global warming, has become one of the major concerns at the international level. With the increasing temperature trend, extreme weather events have become more frequent in many world regions [1].

GHGs are essential components for maintaining the conditions for survival, as well as producing an atmospheric layer that protects against the direct UV rays’ impact on the Earth [2]. At the same time, they are the highest contributors to global temperature augmentation. Since industrialization, an enormous quantity of GHGs have been produced by anthropogenic activities. For example, in 2018, GHG emissions were 41% higher than in 1990 [3].

The principal anthropic sources are transportation (especially from fossil fuels burning), electricity production, agriculture, land use, and forestry [4,5]. The U.S. Inventory [6] indicates that, in 2021, in the USA, the GHG volume exceeded 6340.2 mil mt of CO_2_ equivalents, which 6% higher than in 2020 but 17% lower than in 2005. The study by Hadipoor et al. [7] found that CO_2_ is one of the primary sources of pollution, indicating the correct trend to control the emissions to reduce the amount of gases released in the air.

According to the Intergovernmental Panel on Climate Change (IPCC) special report on climate change, the global temperature has been augmented by 0.8–1.2 °C with respect to the pre-industrial level. It is predicted to rise by 1.5 °C by 2030 and about 3 °C by 2100 if the emissions rate is the same [8].

Some GHG emissions are removed by natural sinks that are globally present. Still, the others (CO_2_, N_2_O, CFCs, HCFCs, PFCs, SF6) can last in the atmosphere for several hundred years, contributing to global temperature increase [9]. Therefore, the GHG emissions must be controlled, and conditions to stimulate their removal through natural sinks (or artificially designed sinks) must be created [3].

According to Naiyer and Abbas [2], although the effect of constant direct exposure of the human body to GHGs appears negligible, their increasing concentration over time is the main cause of different human illnesses. The most affected systems of the human organism are the respiratory system (provoking respiratory acidosis) [10,11], the cardiovascular system [12], the immune system [13,14], the digestive system, and CNS (affecting the brain cells, which may lead to memory loss) [14]. Measuring the impact of GHGs on human health and its quantification are aspects that must be clearly established at the global level [2].

In the context of climate change, the Paris Agreement in 2015 [15] set the goal of limiting the temperature increase, at the global scale, under 2 °C by 2050–2100. The European Union (EU) aims to achieve net-zero emissions by 2050. Therefore, many countries developed renewable energy sources for producing energy from non-pollutant sources. The United Kingdom is working to enhance the removal potential of natural sinks [16]. India (the world’s third largest contributor of GHGs) aims to reduce GHG emissions by 33–35% by 2030, compared to 2005. It also implements measures to generate 40% of electricity from renewable or nuclear sources by 2030 [17]. In the effort to reduce the effects of atmospheric pollution on the climate, in 2020, the European Union achieved a more than 30% emission decrease with respect to the levels from 1990 [18].

Controlling and monitoring the emission sources, understanding the processes produced in the atmosphere by gases’ reactions, and forecasting the effects are essential before taking measures for GHG reduction. Atmospheric conditions and hydro-meteorological variables [19,20,21] influence the gas dissipation and the apparition of secondary products.

Given the importance of the above topic and international concerns, different articles proposed models, especially for estimating GHG emissions from transportation [22,23,24,25]. Alhindawi et al. [23] considered the ratio of vehicle–kilometer, by mode, to the number of transportation vehicles for six transportation modes. They proposed multivariate regression and double exponential smoothing models to forecast GHG emissions. Güzel and Alp [24] utilized the Integrated MARKAL-EFOM System (TIMES) and an economic model for the same goal in three scenarios. The CRTEM/HBEFA-China can be employed to compute future emission scenarios; a software package integrated into CRTEM/HBEFA-China was also developed [25].

In the global context, there is interest in maintaining a decreasing GHG emission trend in Europe and determining the countries that should be sustained to reach the imposed levels. The present study analyzes the total GHGs trend in the UE_27 and some associated members (Switzerland, Norway, and Iceland) from 1990 to 2021. It also groups the series into different clusters using the k-means algorithm and hierarchical clustering. Additionally, two types of series that describe the evolution of GHGs in the EU are proposed. The first is the Regional series (RegS), which is computed using series from selected countries. The second one is the Representative Temporal series (TempS), which is built by selecting specific years after clustering the annual GHG series and applying an original algorithm [5,15].

## 2. Materials and Methods

### 2.1. Data Series

**Figure 1:**
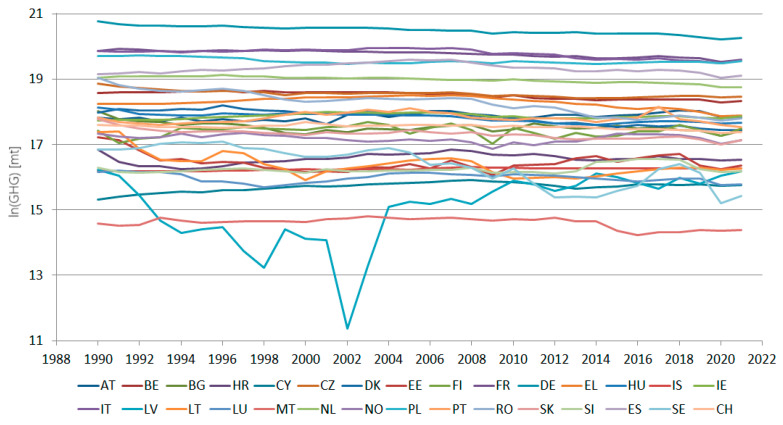
The total CHG series (mt equivalent CO_2_), on a logarithmic scale, in 30 European countries from 1990 to 2021.

The studied data series consists of the total GHG net emissions (in mt CO_2_ equivalent), reported from 1990 to 2021, by the EU-27 countries and three associated members (Switzerland, Norway, and Iceland) to the United Nations Framework Convention on Climate Change. They are retrieved from [26]. Figure 1 presents the studied series, in logarithmic scale, for image clarity.

### 2.2. Methodology

The first step was to test the null hypothesis that there is no monotonic trend against the hypothesis and that such a trend exists for all series. The Mann–Kendall test (MK) [27] was utilized, followed by Sen’s slope procedure [28] when the MK test rejected the null hypothesis. In such a case, a nonparametric linear slope is determined. The procedure is applied for the series registered in each country, the total series (computed by summing up the values recorded in all the countries), and each annual series formed by the values recorded in a specific year in all countries.

In the second stage, the series are grouped in clusters, based on the k-means algorithm [29] and hierarchical clustering [30], to determine the countries with similar pollution levels. The silhouette [31], the elbow–knee method [32,33], and the gap statistics [34,35] were utilized to select the optimal number of clusters, k. The ratio between clusters' sum of squares and the total sum of squares (BSS/TSS) is computed to determine the best clustering in the k-means algorithm. The higher the ratio, the better the clustering. Various methodologies are employed to choose k, so the results of the three algorithms are sometimes different. Therefore, in the present study, we explore the various situations.

Hierarchical clustering provides a dendrogram, showing the hierarchy of the series, which can be assessed and built using the matrix of the distances (Euclidean, Hamming, Manhattan, Cambera, Jaccard, etc.) between the elements (observations, series) that will be grouped. Based on the distance matrix, the similarities between the elements can be assessed by the “average”, “complete”, “median”, “ward.D”, and “ward.D2” methods. Here, we employed the first and last methods. In the average measure, the mean distance between the observations in each group is weighted by the number of observations in each cluster. In the “ward.D2”, the sum of squared errors is minimized, with the clusters being combined based on smaller distances between groups.

To choose between the “average” and “ward.D2”, the cophenetic correlation coefficient [36,37] was utilized. Values above 0.9 show a very good performance, the coefficients in the interval 0.8–0.9 indicate a good performance, and values under 0.8 prove a poor clustering quality. Bootstrapping (resampling from the data set and rerunning the algorithm) is done, and the average Jaccard measures are computed to check if the clustering algorithm provides a good representation of the groups in the studied data set. If they are greater than 0.85 (in the interval 0.6–0.85), the cluster is highly stable (stable). Values less than 0.6 indicate the cluster’s instability [38]. The advantages/disadvantages of k-means and hierarchical clustering are discussed in [39].

In the last stage, the RegS is built using the series recorded in each country (30 series) by the following procedure [15,40].

1. Find the number of clusters for performing the clustering algorithms.
2. Perform the k-means and hierarchical clustering for grouping the countries. Choose the best clustering using the criteria explained above.
3. Select the cluster formed by the highest number of countries, as denoted by Cl_max_. If many clusters have the same largest number of elements, Cl_max_ is that with the highest separation distance from the others and the lowest between the internal members [41].
4. Build the Regional series by averaging the corresponding values of the series in Cl_max_. Thus, the value for the year j is the average of the values recorded in the year j in the countries from Cl_max_.
5. Compute the modeling errors as differences between the recorded values and those of the Regional series.
6. Determine the goodness-of-fit of the Regional series by computing the mean absolute percentage error (MAPE).

The same procedure is applied to the 32 annual series to determine the TempS, with each containing 30 values (reported by a different country during a specific year.

**Figure 2:**
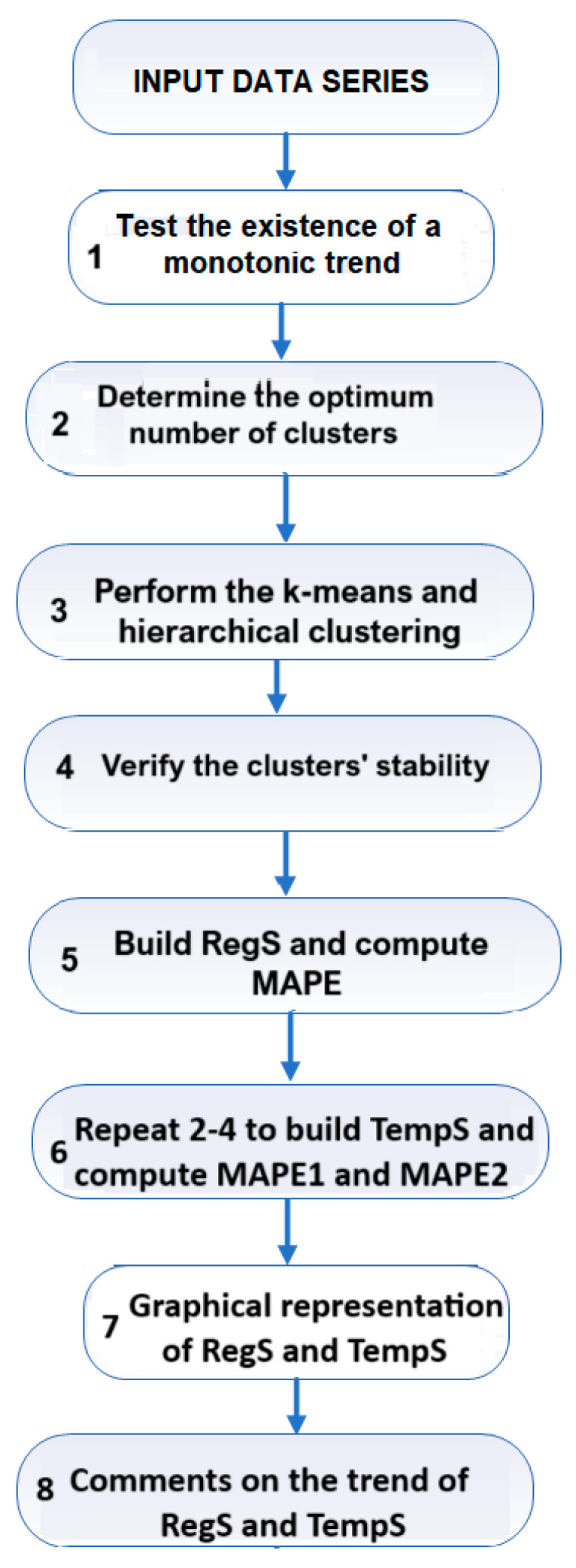
Flowchart of the work.

The flowchart of the work is presented in Figure 2.

The R 4.3.2 software (https://www.r-project.org/, accessed on 21 July 2023) was utilized to conduct the study.

## 3. Results and Discussion

In the following, we shall use the abbreviations of the countries names, according to the international conventions, as follows: Austria (AT), Belgium (BE), Bulgaria (BG), Croatia (HR), Cyprus (CY), Czech Republic (CZ), Denmark (DK), Estonia (EE), Finland (FI), France (FR), Germany (DE), Greece (EL), Hungary (HU), Ireland (IE), Island (IS), Italy (IT), Latvia (LV), Lithuania (LT), Luxembourg (LU), Malta (MT), Netherland (NL), Norway (NO), Poland (PL), Portugal (PT), Romania (RO), Slovakia (SK), Slovenia (SI), Spain (ES), Sweden (SE), and Switzerland (CH).

Analyzing the total annual GHGs series recorded for the EU-27 (obtained by summing up the values recorded in all the EU countries during 1990–2021), two subperiods are determined—before 2002, with a logarithmic trend with the equation:

(1) $$y_{t}=-2\times 10^{8}\ln\left(t\right)+5\times 10^{9}\;(R^{2}=0.850)$$

and after 2003, with a linear trend with the equation:

(2) $$y_{t}=-6\times 10^{7}t+4\times 10^{9}\;\left(R^{2}=0.915\right),$$

where *t* is the time, and $y_{t}$ is the value of the series at the moment *t*.

Overall, the trend of the total GHGs series during 1990–2021 is decreasing.

### 3.1. Building the Regional GHGs Series

Table 1 contains the *p*-values associated with the MK test for each country’s total GHGs series recorded from 1990 to 2021. When the p-value is less than 0.05 (the significance level), the *p*-value is followed (in the brackets) by the sign plus or minus if the slope determined by Sen's method is positive or negative, respectively. Out of 30 countries, the null hypothesis could not be rejected for 9. A negative trend has been determined for 17 series, whereas the estimated total GHGs series trend is positive for only 4 countries (AT, CY, IS, and LV).

**Figure 3:**
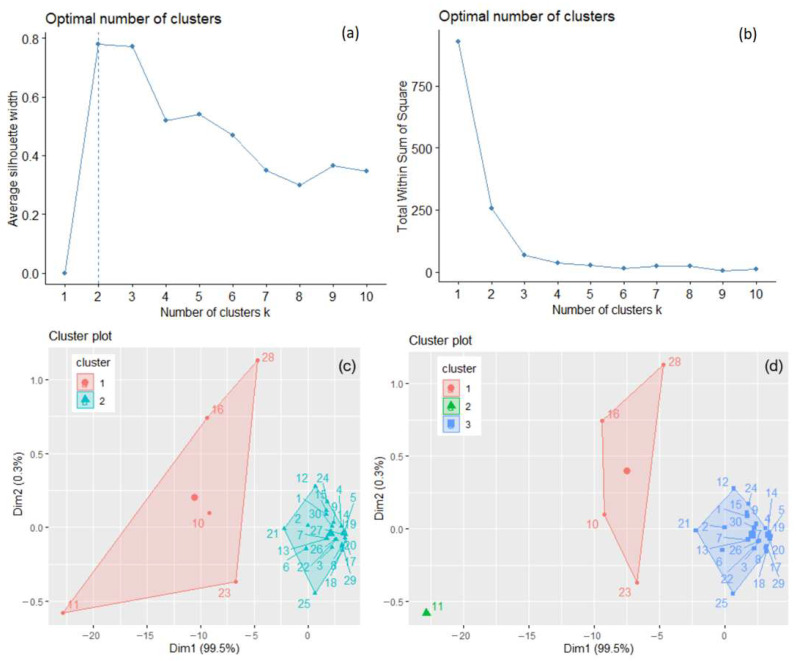
The optimal number of clusters determined for the total GHG series, recorded in 30 countries by the silhouette method (**a**) and the elbow–knee method (**b**). The clusters of the countries are determined by k-means with *k* = 2 (**c**) and *k* = 3 (**d**).

The optimal number of clusters used in the k-means algorithm was determined to be two by the silhouette method, three by the elbow–knee method (Figure 3a,b), and one by the gap statistics. It is known that, when some clusters are close to each other and another one is far from them, the gap statistics can underestimate the value of *k* [35]. Therefore, the option of a single cluster was removed, and the analysis was performed with two and three clusters. Finally, the best solution was chosen.

The clusters that result when applying the k-means algorithm with *k* = 2 and *k* = 3 are presented in Figure 3c,d. When *k* = 2, the within-cluster sum of squares is 206.439 and 49.079, respectively, with the ratio BSS/TSS = 72.5 %. When *k* = 3, the within-cluster sum of squares, by cluster, is 13.321, 0.000, and 49.079, respectively, with the ratio BSS/TSS = 93.8 %. Figure 3d shows a better separation of the clusters (lower values of the within sum of squares, by cluster, and a significantly higher BSS/TSS ratio). To confirm the results, hierarchical clustering was applied for both values, with “ward.D2” and “average” methods. The highest cophenetic coefficient (0.956) was obtained for *k* = 3 clusters and the “average” method, indicating good clustering. After bootstrapping, the obtained average Jaccard values and corresponding instabilities were 0.632 (0.368), 0.742 (0.252), and 0.964 (0.000) for clusters 1, 2, and 3, respectively, indicating the clusters’ stability.

The k-means and hierarchical clustering provided the same groups of countries based on the total emitted GHGs.

**Figure 4:**
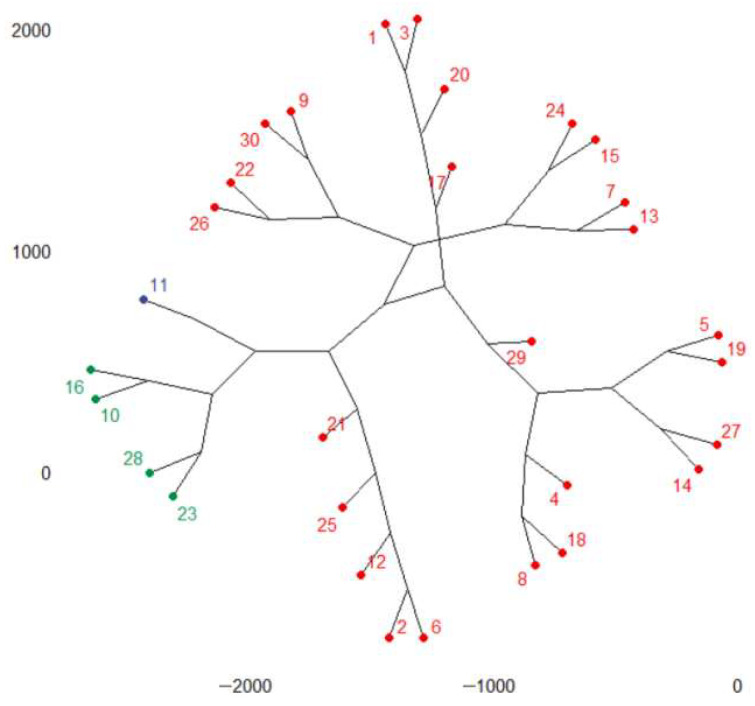
The phylogenic dendrogram for the total GHGs series. Cluster 1: FR (10), IT(16), PL(23), ES(28); cluster 2: DE(11); cluster 3: the rest of the countries.

The phylogenic dendrogram for *k* = 3 is presented in Figure 4. According to Figure 4, France, Italy, Poland, and Spain belong to the first group, Germany belongs to the second group, and the rest of the countries are in the third cluster. Germany is the county with the highest emissions.

In the countries from the second group, the recorded emissions are above 228 mil mt and below 565.4 mil mt CO_2_ equivalent. In the countries from the third group, the maximum recorded value of GHGs was 228,533,250 mt CO_2_ equivalent.

Given the above results, the regional series was built using the series from the third cluster. The MAPE (Table 2) goodness-of-fit indicator is a non-dimensional indicator that gives a better image of the fitting quality than the dimensional indicators and permit comparisons between different data series. The lower the MAPE is, the better the fitting is. The fitting results are good for most countries. The series in the first and second clusters are well estimated. Very high values of MAPE are recorded for some countries belonging to the last group, which have very low emissions.

### 3.2. Building the Representative Temporal Series

According to Table 3, which contains the *p*-values in the MK test for the annual total GHG series, the null hypothesis could not be rejected for all these series, so one cannot assume the existence of a monotonic trend of annual series.

**Figure 5:**
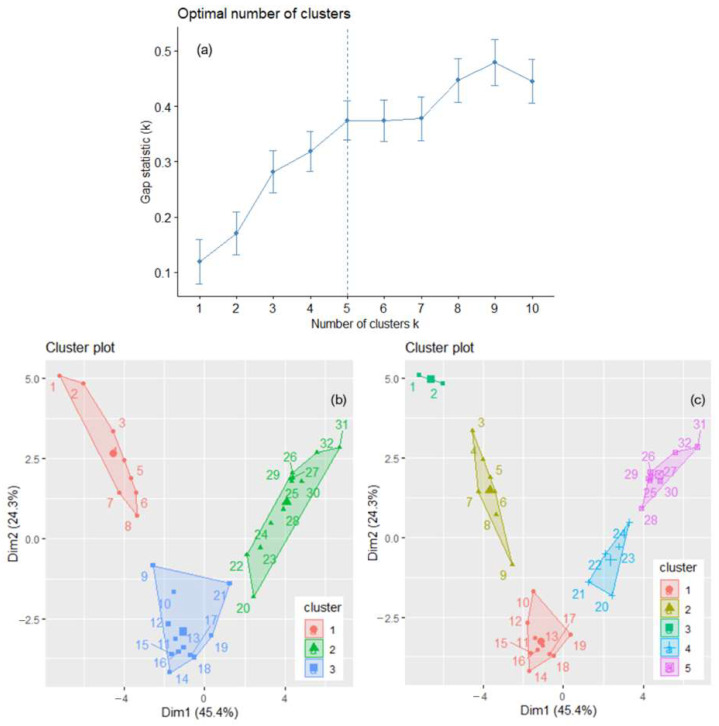
(**a**) *k* = 5, indicated by the dotted vertical line, is the optimum number of clusters found by the gap statistics. The clusters of the annual GHGs series are determined by k-means with (**b**) *k* = 3 and (**c**) *k* = 5. The years are numbered from 1 to 32, corresponding to the years from 1990 to 2021.

The optimal number of clusters for the annual total GHGs series is either three—determined by the silhouette and elbow–knee methods—or five—detected by the gap statistics (Figure 5a). Therefore, the analysis was done for *k* = 3 and *k* = 5. The clusters obtained by the k-means algorithm for the annual GHGs series are presented in Figure 5b,c. The years are from 1 (1990) to 32 (2021). BSS/TSS is 59.9% when *k* = 3 and 76.3% when *k* = 5. When *k* = 3, 1990–1997 (1–8 in Figure 5b) belong to the first cluster, 1998–2008 and 2010 are in the second, and 2009 and 2011–2021 belong to the third.

The within-cluster sum of squares by cluster is, respectively, 119.414, 106.360, and 147.550 (and the BSS/TSS = 59.9%). When *k* = 5 (Figure 5c), the clusters are formed by the years 1990 and 1991 (cluster 3), 1992–1998 (cluster 2), 1999–2008 (cluster 3), 2009–2013 (cluster 4), 2014–2021 (cluster 5). The cophenetic coefficient was 0.8119 (0.7163) when using the “average” (“ward.D2”) method in the hierarchical clustering. Therefore, the first one was employed to build the clusters.

**Figure 6:**
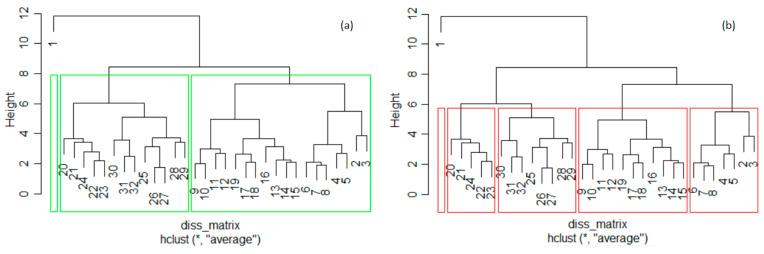
Dendrogram of the annual total GHGs series and clusters determined by hierarchical clustering with (**a**) *k* = 3 and (**b**) *k* = 5. The years are numbered from 1 (1990) to 32 (2021).

The dendrograms (Figure 6) do not confirm the clustering by the k-means. In both dendrograms, the first years form a separate cluster. Still, for *k* = 3, the second cluster in the dendrogram includes the years from the second cluster in the k-means and 2010. The first cluster, except 1990, and the third one in the k-means are merged to obtain the third cluster in Figure 6a.

For *k* = 5, 1991 (year 2 from cluster 3 in Figure 5c) forms a single cluster in the hierarchical clustering (Figure 6b, the left-hand-side cluster), and 1991 was included in the cluster containing 1992–1997 (the right-hand cluster in Figure 6b). The year 1998 (year 9 from cluster 2 in Figure 5c) was moved to the fourth cluster (from left to right in Figure 6b) together with 1999–2008.

The fourth (and fifth) cluster in k-means coincides with the third (and second) in Figure 6b. Thus, there are only a few differences between the clusters provided by the k-means and hierarchical clustering when *k* = 5. After bootstrapping, the average Jaccard values and corresponding instabilities in the k-means clustering with *k* = 3 are 0.824 (0.070), 0.847 (0.166), and 0.988 (0.020), respectively, indicating high stability. For *k* = 5, lower stabilities were obtained. Therefore, the best clustering of the annual total of GHG series was obtained with *k* = 3 (Figure 6a). Therefore, the series that will participate in building TempS belongs to the cluster with the highest ratio of BSS/TSS in the *k*-means algorithm: the third cluster.

Table 4 provides the MAPE in this case (MAPE 1) and compares them with those obtained when the representative temporal series would be computed using the elements from the second cluster (MAPE2). The values of MAPE1 vary between 4.134 and 40.619 for all years but 2012. MAPE2’s variation interval is 5.310–60.698, except for 2012 when it is 312.412 because of the very low value recorded in Latvia (109.406 mt CO_2_ equivalent). The average MAPE is significantly lower (21.129 compared to 30.661) when building the TempS with the elements in the third cluster.

### 3.3. General Comments

**Figure 7:**
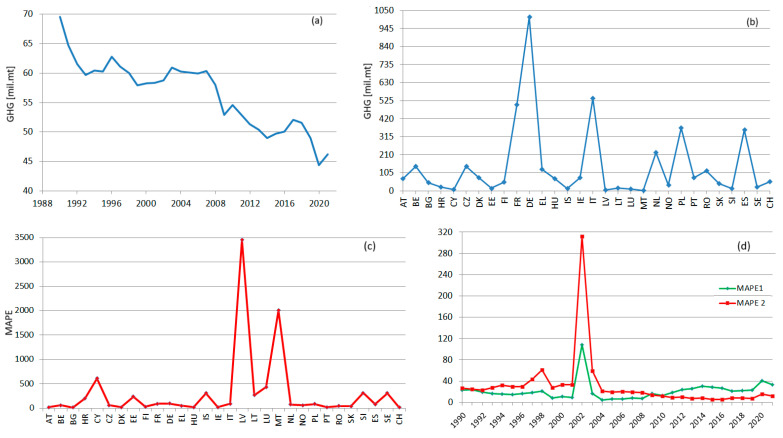
(**a**) RegS and (**b**) TempS; (**c**) MAPE for RegS; (**d**) MAPE 1 and MAPE 2 for TempS.

The charts of RegS and TempS are shown in Figure 7, together, with the corresponding MAPEs. None of RegS and TempS are linear. An overall decreasing trend can be emphasized for RegS (Figure 7a), with slight subperiods of augmentation followed by periods of abrupt decrease. The presence of decrement periods is due to the existence of series with an increasing tendency or periods of variation around a particular value, followed by decay periods. Still, the decrease in RegS from 1990 to 2021 is significant.

In the case of RegS, the inhomogeneities in the GHG emissions are reflected in high MAPE for the lowest producer countries, which might be considered outliers (the picks in Figure 7c).

The TempS emphasizes the existence of high emissions producers, which are constantly the same during the study period (DE, FR, IT, SE, PT). When analyzing the MAPE1, remark that, during 1990–1998 and 2009–2011, the values are in the same range, whereas for 1999, 2001, and 2004–2008, the values are much lower; the highest values correspond to 2012–2021. These ranges of values are related to the slight variations of the annual series.

The values of MAPE 2 are significantly higher than those of MAPE 1 between 1990 and 2009, reflecting a worse fitting of TempS. After 2012, MAPE 2 becomes lower than MAPE 1. Given the high variations in the previous period, the average MAPE1 is about 1.5 lower than the average MAPE 2. These values are influenced by the values of the series that participate in building TempS. An idea that will be explored in the future is fitting TempS using subseries that better fit the set of studied series on different subperiods.

**Figure 8:**
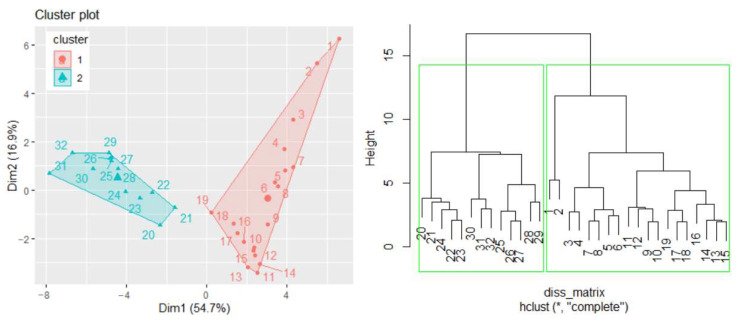
(**left**) Clusters of the GHG per capita series for building TempS and (**right**) the dendrogram.

A similar study can be performed considering other variables, such as population or GDP, when the studied series will be formed by the annual GHG emissions in mt CO_2_ equivalent/per capita and GHG emissions in mt CO_2_ equivalent/per GDP, respectively. In the first case, when determining the TempS, the best number of clusters is two, and the hierarchical clustering confirms the results of the k-means algorithm (Figure 8). The cluster that participates in building TempS contains the series recorded in the first 19 years (the first cluster in Figure 8(left)). A detailed study will be presented in another article.

## 4. Conclusions

This work analyzed the total GHG series recorded in 30 European countries to emphasize the series trend. The RegS and TempS were also built by an original algorithm. The series that participated in creating these representative series were selected after determining the cluster with the highest number of elements. When using the optimal value provided for k (for running the k-means algorithm) by different criteria differs, the separation and stability criteria are crucial for choosing the correct number of clusters to cluster the series. Selecting the optimum number of groups (*k*) is essential for fitting RegS and TempS since the estimation accuracy is influenced by the series values participating in the process.

There are notable differences between the GHG emissions in different countries. Germany is the highest pollutant producer, and small countries, such as Malta, are the lowest. This situation and the increasing tendencies of GHG series in some countries contribute to the low fitting quality of the recorded series in the mentioned countries.

The data series used are trustworthy. Even if some reporting errors are possible, when one is interested in the regional or temporal evolution of GHG emissions in Europe, the trend shown by the presented models is the same (only the accuracy is lower). The main advantage of the proposed models is that they give an image of GHG's spatial and temporal evolution over a region. They can be built without restrictions related to the territory or specific requirements on the series distributions.

In a future study, we intend to extend the analysis to the specific GHGs (CO_2_, CH_4_, N_2_O, fluorinated gases, etc.) and incorporate other variables, such as GDP and population.

## Figures and Tables

**Table 1:** The *p*-values in the MK test for the total GHGs per country.

**Country**	**AT(1)**	**BE(2)**	**BG(3)**	**HR(4)**	**CY(5)**	**CZ(6)**	**DK(7)**	**EE(8)**	**FI(9)**	**FR(10)**
*p*-val	0.002 (+)	0.000 (-)	0.002 (-)	0.116	0.000 (+)	0.000 (-)	0.000 (-)	0.615	1.000	0.000 (-)
**Country**	**DE(11)**	**EL(12)**	**HU(13)**	**IS(14)**	**IE(15)**	**IT(16)**	**LV(17)**	**LT(18)**	**LU(19)**	**MT(20)**
*p*-val	0.000 (-)	0.095	0.000 (-)	0.000 (+)	0.446	0.002 (-)	0.000 (+)	0.001 (-)	0.039 (-)	0.249
**Country**	**NL(21)**	**NO(22)**	**PL(23)**	**PT(24)**	**RO(25)**	**SK(26)**	**SI(27)**	**ES(28)**	**SE(29)**	**CH(30)**
*p*-val	0.000 (-)	0.012 (-)	0.000 (-)	0.961	0.000 (-)	0.000 (-)	0.168	0.961	0.000 (-)	0.001 (-)

**Table 2:** MAPE (%) in modeling the Regional Series GHGs series.

**Country**	**AT(1)**	**BE(2)**	**BG(3)**	**HR(4)**	**CY(5)**	**CZ(6)**	**DK(7)**	**EE(8)**	**FI(9)**	**FR(10)**
MAPE	18.636	57.957	14.417	198.795	612.099	59.921	18.428	237.288	28.681	88.145
**Country**	**DE(11)**	**EL(12)**	**HU(13)**	**IS(14)**	**IE(15)**	**IT(16)**	**LV(17)**	**LT(18)**	**LU(19)**	**MT(20)**
MAPE	94.240	47.278	16.517	308.665	20.147	88.265	3454.276	265.811	434.350	2008.650
**Country**	**NL(21)**	**NO(22)**	**PL(23)**	**PT(24)**	**RO(25)**	**SK(26)**	**SI(27)**	**ES(28)**	**SE(29)**	**CH(30)**
MAPE	73.837	59.746	85.186	17.727	45.936	38.440	311.552	81.317	309.084	11.831

**Table 3:** The *p*-values in the MK test for the annual total GHGs series.

**Year**	**1990**	**1991**	**1992**	**1993**	**1994**	**1995**	**1996**	**1997**	**1998**	**1999**	**2000**
*p*-val	0.695	0.643	0.669	0.7219	0.695	0.775	0.748	0.775	0.721	0.695	0.803
**year**	**2001**	**2002**	**2003**	**2004**	**2005**	**2006**	**2007**	**2008**	**2009**	**2010**	**2011**
*p*-val	0.831	0.831	0.775	0.775	0.775	0.643	0.568	0.593	0.544	0.669	0.498
**year**	**2012**	**2013**	**2014**	**2015**	**2016**	**2017**	**2018**	**2019**	**2020**	**2021**	
*p*-val	0.498	0.498	0.392	0.412	0.412	0.521	0.593	0.454	0.412	0.373	

**Table 4:** MAPE (%) in building TempS for the total GHGs series.

**Year**	**1990**	**1991**	**1992**	**1993**	**1994**	**1995**	**1996**	**1997**	**1998**	**1999**	**2000**
MAPE 1	23.274	23.713	19.072	16.316	15.960	14.861	16.195	18.674	21.479	8.117	10.633
MAPE 2	26.432	25.038	23.194	27.968	31.817	29.199	29.481	43.373	60.698	27.782	32.982
**year**	**2001**	**2002**	**2003**	**2004**	**2005**	**2006**	**2007**	**2008**	**2009**	**2010**	**2011**
MAPE 1	8.761	108.174	16.940	4.134	6.244	6.053	8.019	7.612	16.825	12.516	17.927
MAPE 2	33.274	312.412	59.004	21.449	19.340	20.283	19.235	18.006	13.577	11.532	9.208
**year**	**2012**	**2013**	**2014**	**2015**	**2016**	**2017**	**2018**	**2019**	**2020**	**2021**	**average**
MAPE 1	23.585	25.538	30.353	28.333	26.579	21.610	21.686	23.361	40.619	32.957	**21.129**
MAPE 2	9.735	7.143	7.873	5.442	5.310	8.093	7.738	7.081	15.797	11.657	**30.661**

## Data Availability

Data will be available on request from the author.
